# Eggshell sourced hydroxyapatite as a bone regenerative material in dentistry – a narrative review

**DOI:** 10.2340/biid.v13.45750

**Published:** 2026-05-18

**Authors:** Konathala SV Ramesh, Mopati Nishanth Reddy Gokul, Penmetsa S Gautami, Naga Venkata SG Sruthima, Pasupuleti Mohan Kumar, Kanakamedala Anil Kumar, Mathala Venkata Lakshmi

**Affiliations:** Department of Periodontics, Vishnu Dental College, Bhimavaram, India

**Keywords:** Bone, bone density, demineralised freeze-dried bone allograft, eggshell-derived hydroxyapatite

## Abstract

Although bone is a dynamic connective tissue with inherent regenerating potential, grafting materials are frequently needed to recuperate shape and function in cases of bone defects. Eggshell-derived hydroxyapatite (EHA) and its nano-form (EnHA) have become viable alternatives because of their availability, affordability, osteoconductivity, biocompatibility, and environmental friendliness.

Human studies evaluating the clinical potential of EHA/EnHA in bone regeneration that were published between 2014 and 2024 were reviewed. A total of seven trials with 112 patients and 88 intervention locations were included in this narrative review. EHA showed promising results in improving bone density, volume, and healing time across a variety of motives including socket preservation, intrabony defects, apicoectomy, and cystic lesions. When paired with platelet-rich fibrin, EnHA consistently demonstrated better healing than ungrafted areas and equivalent outcomes to conventional grafts, such as demineralised freeze-dried bone allograft and synthetic hydroxyapatite. Significant improvements in bone density and the production of new trabecular bone were confirmed by radiographic and histomorphometric investigations.

The therapeutic viability of materials generated from eggshells is further supported by their high calcium content, non-cytotoxic profile, and efficient high-temperature sterilising. However, there are drawbacks, such as inconsistent resorption rates, a dearth of long-term randomised controlled studies, and a lack of established preparation procedures. Large multi-centre studies, long-term result validation, and manufacturing process optimisation should be the main areas of future study. However, with the existing literature, EHA and EnHA are found to be affordable, biocompatible, and sustainable substitutes for conventional graft materials in bone repair and regeneration in dentistry.

## Introduction

Bone is a dynamic connective tissue, and bone regeneration is a complex, well-orchestrated physiological process that has the intrinsic capacity to repair in response to injury. Bio-based materials have gained popularity, with a focus on exploring their potential in biomedical applications [1]. These materials are advantageous for a variety of applications, including dentistry, because of their distinctive characteristics of non-toxicity, plentiful availability, biological suitability, and biological decomposition [2].

More than 50 bone replacements have been crafted since Van Meekren’s initial bone transplant in 1668, but none have found extensive therapeutic use. The perfect bone substitute should be ample, mechanically strong, biocompatible, osteoconductive or osteoinductive, and at a fair price [3]. Although autografts are considered the best option, their scarcity, donor site morbidity, and hazards such as nerve injury, infection, haemorrhage, scarring, loss of function, lengthy operation, and expensive expenses, however, restrict their utilisation [3, 4]. To tackle these problems, new materials have been created, but their effectiveness is hampered by the scarcity of viable osteoblasts [4].

Bovine grafts and hydroxyapatite (HA) are important xenograft materials prized for their low resorption rates and osteoconductive properties [3]. The main inorganic component of bone and teeth, HA is composed of calcium phosphate, which is the main inorganic component of bone and teeth, and comes from a variety of sources, including people, animals, coral, and eggshells [5]. It repairs bone defects and is progressively replaced by new bone; the rate of replacement is determined by the rate at which HA degrades [5]. Bovine-derived HA, for instance, has been shown to stay in the human maxillary sinus for more than 10 years after sinus lift without causing foreign body responses, demonstrating the long-term persistence of slow-resorbing HA [6].

Hydroxyapatite’s strong bioactivity allows it to quickly form an association with bone by concentrating fibronectin on its surface [2]. It is often used in treatments including ridge augmentation, defect grafting, sinus grafting, and socket preservation [2]. Instead of commercial grafts, eggshell-derived hydroxyapatite (EHA) has recently demonstrated better osteoconductive qualities and increased bone healing in rats. EHA is a viable material for regenerative bone grafting because of its hydrophilicity, ease of handling, biocompatibility, and absence of disease transmission hazards [2, 7]. This narrative review evaluates the effectiveness of employing EHA or its nano-form (EnHA), and whether or not it produces positive results in terms of bone regeneration.

## Methodology

This article employed a narrative review methodology. By gathering, evaluating, and summarising all of the research on a specific topic, a narrative review seeks to lessen the bias of study findings. Keywords like “bone loss” OR “defect” OR “bone defect” AND “autograft” OR “allograft” OR “bone substitute” AND “xenograft” AND “eggshell” AND “hydroxyapatite” were used to search major scientific research databases such as PubMed and Cochrane.

**Figure 1:**
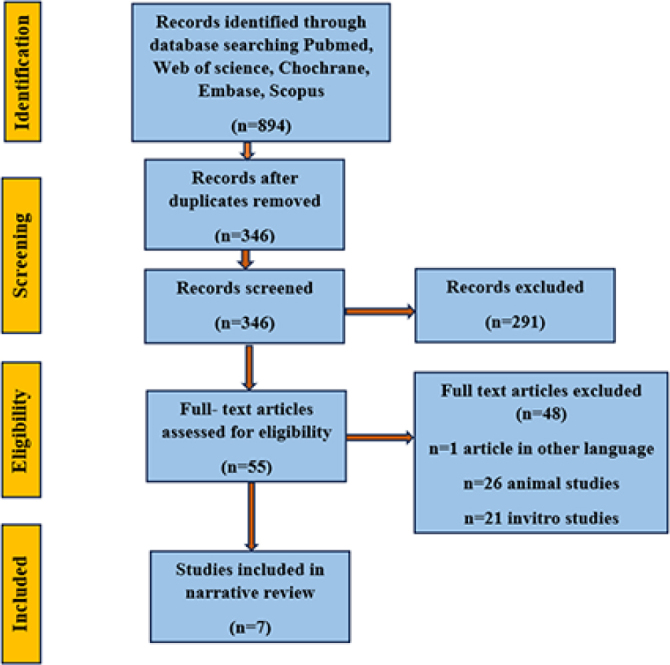
PRISMA flow chart.

After duplicates were eliminated, the 894 items that the computerised search produced were narrowed down to 346. A manual search turned up no more articles. A total of 291 records were excluded after titles and abstracts were screened. Of the remaining studies, the full texts of 55 papers were obtained, of which 48 studies were excluded. The full texts of remaining seven publications underwent methodical, quality-based analysis (Figure 1, Table 1). Only human studies and articles published from inception to 2024 were included.

**Table 1:** Characteristics of the clinical trials included.

Author, year [ref]	Type of study	Procedure	Sample size	Intervention	Comparison	Outcomes
Kattimani et al. (2019) [20]	RCT, split mouth	Socket preservation	12	EnHA	Placebo, no graft	Increased bone density after 6 months
Nainoor et al. (2024) [3]	RCT, split mouth	Socket preservation	20	EnHA	DFDBA	After 6 months, bone density increased in both groups
Kattimani et al. (2019) [19]	Prospective study	Socket preservation	11	EnHA+ PRF	Not specified	Increased bone density in 24 weeks
Kattimani et al. (2014) [29]	Preliminary study	Maxillary cystic defects	8	EHA	SHA	Increased bone density after 3 months
Kattimani et al. (2016) [7]	RCT, split mouth	Cystectomy	20	EHA	SHA	Increased bone density after 6 months
Kattimani et al. (2019) [30]	Prospective clinical study	EHA after cystectomy	20	EHA	Not specified	Uneventful healing of bone occurred in 8 weeks
Vani et al. (2023) [27]	Interventional RCT	Intrabony defects	21	1. EnHA graft+Periosteal pedicle membrane 2. EnHA as a graft	Open flap debridement	Increased bone density in both groups at 6 months

RCT: Randomized Controlled Trial; EnHA: Eggshell-derived nano-hydroxyapatite; PRF: Platelet rich fibrin; EHA: Eggshell-derived hydroxyapatite; DFDBA: Demineralized freeze dried bone allograft; SHA: Synthetic hydroxyapatite.

A total of seven publications including 88 intervention sites and 112 patients were included. By assessing evaluations from the baseline and follow-up procedures following the use of eggshell sourced hydroxyapatite as bone graft material, the assessment of the primary outcome aimed to determine whether there were any significant improvements in alveolar bone dimensions, density, and healing time.

## Discussion

Recycling chicken eggshells benefits the environment by reducing waste and providing raw materials for nanomaterials development [8]. The majority (94%) of eggshells is made of calcium carbonate, with remainder consisting of organic matter (including insoluble proteins), magnesium carbonate, and calcium phosphate (4%, 1%, and 1%, respectively) [9, 10]. Other natural sources of hydroxyapatite, such as corals, cuttlefish, and oyster shells, as well as fish and cattle bones, have also been utilised to create biomaterials. But unlike eggshells, these sources are susceptible to depletion if they are continuously harvested, especially for slow-growing corals [8, 11].

While comparing the hydroxyapatite (HAp) made from eggshell to HAp made from other sources, the former has shown better outcomes in terms of density and hardness. The biological suitability of eggshell based HAp has been confirmed by a cytotoxicity test conducted utilising osteoblast cell culture [12]. It demonstrates that eggshell based HAp is noncytotoxic and promotes osteoblast cell attachment [12, 13]. The calcination temperature for eggshell-based HAp ranges up to 900°C. At this temperature, all disease-transmission concerns are eliminated by eliminating bacteria that can affect patients [12, 14].

In both human and animal models, eggshells have demonstrated potential as a bone regenerative material due to their high calcium content. EHA and EnHA have been shown to be very successful in promoting bone repair, providing a viable substitute for tissue regeneration. The results indicate eggshell-based materials, particularly in the nano-form, enhanced the Guided Bone Regeneration (GBR) process [15–18].

### Socket preservation

In a prior study, bone width and radiographic density were measured at 1, 12, and 24 weeks after 23 sockets and 11 patients had been grafted with EnHA and coated with a platelet rich fibrin (PRF) membrane. Over the course of 24 weeks, the results revealed a substantial increase in bone density (*p* < 0.05), with 26.09% of sockets having a ground-glass look and 73.91% having a trabecular pattern. Micro-CT verified the creation of new trabecular bone, whereas histomorphometric analysis showed Grade-3 bone in 56.52% sockets, Grade-2 in 13.04% sockets, and Grade-1 in 8.70 sockets%. However, the absence of a control group was a major limitation [19].

In a split mouth randomized control trial (RCT),12 patients (24 extraction sites) were grafted with EnHA (12 sites) and the remaining sites with no graft after surgical removal of bilateral mandibular molars. Radiographic bone density was assessed at baseline, first, third, and sixth month; the analysis showed a significant increase in bone density in the graft group when compared to control group (*p* < 0.05), indicating complete bone healing in the graft group at the 3-month and 6-month recalls [20].

In a split mouth study, 40 extraction sites were equally divided and grafted with EnHA+PRF and the remaining sites with Demineralized Freeze-Dried Bone Allograft (DFDBA)+PRF. The dissimilarity in mean bone density involving the EnHA group and the DFDBA group was not statistically significant immediately post-grafting (*p* > 0.05), after the first month (*p* > 0.05), as well as after fourth months (*p* > 0.05). A significant difference was observed within the group at different intervals, showing an improvement in bone density [2].

Because of its high water permeability, low toxicity, stability, affordability, and ease of application, eggshell membrane has emerged as a potential biomaterial in recent years. In comparison to only a PRF membrane, eggshell membrane dramatically increases bone density and socket volume over a 6-month period while decreasing bone resorption [21]. Similarly, several authors determined that it contains excellent regenerative and osteogenic potential [22–27].

### Intrabony defects

A study found that, at 6 months, the pedicle membrane with EnHA group and the EnHA alone group had significantly greater bone density and bone fill than the open flap debridement group in intrabony defects [28]. Opris et al. reported eggshell to be an attainable filler material for bone regeneration treatments, which may be utilised effectively either alone or in conjunction with PRF for alveolar preservation procedures [4]. Similarly, Wardana et al. found that, particularly after 6 months, eggshell produced full bone regeneration or improved bone density in proportion to the bovine group in the transplanted tooth region [29].

### Maxillary cystic defects

After apicoectomy and/or cystic enucleation, eggshell was employed as a bone graft material in eight maxillary bone defects. By the eighth week, there was a noticeable increase in bone regeneration, with just a small amount of bone regeneration occurring in non-grafted locations [30].

### Apicectomy

EHA dramatically increased bone regeneration between weeks 4 and 8, resulting in smooth wound healing and trabecular bone apparent by the 12^th^ week [31]. In contrast, when comparing EHA with synthetic hydroxyapatite (SHA) at 1 and 6-month intervals, there was no bone regrowth. This might be because graft particles resorb either early or late [7].

However, the synthesis route and processing parameters of EHA play a critical role in determining its physicochemical and biological properties. Common synthesis techniques include wet chemical precipitation, hydrothermal processing, and calcination-based methods. Calcination temperatures ranging from 800°C to 900°C have been reported to influence crystallinity, phase purity, and particle size while ensuring sterilisation and elimination of organic contaminants. Higher calcination temperatures generally increase crystallinity but may reduce resorption rates, whereas lower temperatures yield poorly crystalline hydroxyapatite with enhanced biodegradability. Nano sized particles produced via hydrothermal or precipitation methods demonstrate superior bioactivity, improved osteoblast response, and faster bone regeneration. However, the absence of standardised synthesis protocols across studies limits direct comparison of functional outcomes. Establishing optimal synthesis parameters is essential for achieving predictable clinical performance [32].

## Conclusion

EHA and its nano version are economical, environmentally benign, and biocompatible substitutes. In a variety of clinical applications, they successfully improve bone density and healing, and their results are on par with those of traditional grafts such as SHA and DFDBA. The comparable efficacy of EHA and EnHA to that of established graft materials, coupled with their non-cytotoxic nature and ability to support osteoblast activity, make them suitable for a wide range of applications including socket preservation, intrabony defect repair, and cystic defect management. Further, their environmentally friendly production from waste resources adds even more value.

### Limitations

Only seven human clinical studies were eligible for inclusion, and several originated from the same research groups, which may introduce publication or investigator bias. The limited number of RCTs and the lack of long-term follow-up restrict the generalisability of the findings. In addition, heterogeneity in study design, defect types, follow-up periods, and outcome assessment methods prevented direct quantitative comparison. The absence of standardised preparation protocols for EHA further complicates interpretation of outcomes.

### Future prospects

To fully realise the promise of eggshell-derived biomaterials in standard clinical practice, future research should concentrate on refining processing methods, identifying optimal therapeutic indications, and proving comparative efficacy with bigger, multi-centre trials.

## Data Availability

All the data related to the current review are available in the manuscript. No additional information is required to be available in other sources.
